# Patients with schizophrenia and bipolar disorder display a similar global gene expression signature in whole blood that reflects elevated proportion of immature neutrophil cells with association to lipid changes

**DOI:** 10.1038/s41398-023-02442-1

**Published:** 2023-05-05

**Authors:** Anja Torsvik, Hans-Richard Brattbakk, Andrea Trentani, Rita Holdhus, Christine Stansberg, Christoffer A. Bartz-Johannessen, Timothy Hughes, Nils Eiel Steen, Ingrid Melle, Srdjan Djurovic, Ole A. Andreassen, Vidar M. Steen

**Affiliations:** 1 NORMENT, Department of Clinical Science, University of Bergen, Bergen, Norway; 2 Dr. Einar Martens Research Group for Biological Psychiatry, Department of Medical Genetics, Haukeland University Hospital, Bergen, Norway; 3 Computational Biology Unit, Department of Informatics, University of Bergen, Bergen, Norway; 4 NORMENT, Division of Psychiatry, Haukeland University Hospital, Bergen, Norway; 5 NORMENT, Institute of Clinical Medicine, University of Oslo, Oslo, Norway; 6 Department of Medical Genetics, Oslo University Hospital, Oslo, Norway; 7 Division of Mental Health and Addiction, Oslo University Hospital, Oslo, Norway

**Keywords:** Clinical genetics, Schizophrenia, Bipolar disorder

## Abstract

Schizophrenia (SCZ) and bipolar disorder (BD) share clinical characteristics, genetic susceptibility, and immune alterations. We aimed to identify differential transcriptional patterns in peripheral blood cells of patients with SCZ or BD versus healthy controls (HC). We analyzed microarray-based global gene expression data in whole blood from a cohort of SCZ (*N* = 329), BD (*N* = 203) and HC (*N* = 189). In total, 65 genes were significantly differentially expressed in SCZ and 125 in BD, as compared to HC, with similar ratio of up- and downregulated genes in both disorders. Among the top differentially expressed genes, we found an innate immunity signature that was shared between SCZ and BD, consisting of a cluster of upregulated genes (e.g.*, OLFM4, ELANE, BPI* and *MPO*) that indicate an increased fraction of immature neutrophils. Several of these genes displayed sex differences in the expression pattern, and post-hoc analysis demonstrated a positive correlation with triglyceride and a negative correlation with HDL cholesterol. We found that many of the downregulated genes in SCZ and BD were associated with smoking. These findings of neutrophil granulocyte-associated transcriptome signatures in both SCZ and BD point at altered innate immunity pathways with association to lipid changes and potential for clinical translation.

## Introduction

Both schizophrenia (SCZ) and bipolar disorder (BD) have high estimated heritabilities and a lifetime risk of about 1–2% worldwide [1–4]. The clinical onset is usually in late adolescence or early adulthood, and these psychiatric disorders constitute a high burden to the patients, their families and society. A diagnosis of SCZ or BD is associated with 8–14 years shorter life expectancy than the average population [5] and an increased risk of cardiovascular disease, respiratory diseases and infections [6]. The disease mechanisms of SCZ and BD are not known in detail, but the two disorders display considerable overlap in genetic risk factors, clinical symptoms, cognitive dysfunctions, and treatment regimens [7–13]. At present, genome-wide association studies (GWAS) have identified common single nucleotide polymorphisms (SNPs) in 287 loci with genome-wide significant association to SCZ [14], whereas 64 such loci have been reported for BD [15]. Additional genetic risk factors include rare genomic copy number variants (i.e. CNVs) and ultra-rare gene-disrupting variants [14, 16]. The genetic findings have pointed at some underlying disease mechanisms, such as alterations in synaptic function and plasticity [17].

Transcriptome analysis constitutes an alternative approach to identify biological disturbances associated with the disorders. Post-mortem brain transcriptome analyses may reveal pathways associated with the pathology of the disease, while ex vivo blood transcriptomics may add knowledge about the course of the disease and treatment response. Gene expression studies from SCZ brain tissue have pointed at changes in pathways related to synaptic function, cell adhesion, and immune processes such as the complement cascade [18–20]. Differential gene expression (DEG) patterns from peripheral blood samples from SCZ, BD, and healthy controls (HC), have displayed changes in pathways related to innate immune response, mitochondrial activity and apoptosis [21–27]. Such findings were reported by Leirer and colleagues in the current largest published global gene expression dataset of whole blood from 131 first-episode psychosis (FEP) and 149 HC [28]. Interestingly, in their microarray-based study they found that positive symptoms correlated with immune pathways while negative symptoms correlated with mitochondrial pathways.

Our aim was to extend the search for differential gene expression signatures in SCZ and BD, compared to HC, based on comprehensive microarray-based transcriptome analysis of whole blood samples and availability of rich phenotype information, to further explore disease- and/or treatment-related biological mechanisms in psychotic disorders.

## Material and methods

### Study design and ethics

The present study combines data from the Thematically Organized Psychosis (TOP) study at the University of Oslo, Oslo University Hospital (http://www.med.uio.no/norment/), and collaborating Norwegian hospitals and a global microarray-based gene expression study on patients with severe mental disorders (incl. many TOP participants). The Regional Committee for Medical Research Ethics and the Norwegian Data Inspectorate approved the study. The Norwegian Directorate of Health approved the biobank. All participants provided written informed consent.

### Clinical assessment protocol for TOP participants

The TOP participants include patients diagnosed with psychotic disorders (SCZ or BD) and Healthy Controls (HC). The clinical assessment protocol has been described earlier [29]. The main inclusion criteria for patients were: (1) meeting the diagnostic criteria for broad schizophrenia or bipolar spectrum disorders according to the Diagnostic and Structural Manual of Mental Disorders, fourth version (DSM-IV, American Psychiatric Association, 2000), (2) no head trauma, neurological or other medical disorder that could influence CNS functioning, (3) estimated IQ above 70, and (4) age 18 to 65 years. HC were randomly selected from the The Norwegian Tax Administration/National Population Registry (http://www.skatteetaten.no/) from the same catchment area as the patients. Inclusion criteria for HC were: (1) no history of severe psychiatric disorder in HC, nor in their first-degree relatives, and (2) no substance/alcohol abuse/dependency.

### Blood sampling and mRNA isolation for the Global microarray-based gene expression study

Blood was collected in Tempus Blood RNA Tubes. Blood sampling was between 7 am and 7 pm, and according to the blood sampling protocol, participants should be fasting.

Total RNA was isolated semi-automated from whole blood using the Applied Biosystems 6100 instrument and Tempus 12-Port Isolation kit, or manually using the Applied Biosystems Tempus Spin RNA Isolation kit (Applied Biosystems, Austin, TX, USA). RNA integrity number (RIN) values were only available for a subset of the samples (*N* = 214), and those samples had a mean RIN value of 8.8 (±1.0). The RNA tubes were stored at −80 °C.

Clinical chemistry parameters were analyzed at the Department of Medical Biochemistry, Oslo University Hospital, Oslo, Norway. Cholesterol (total, HDL and LDL) and triglyceride were directly measured on an Integra 800 instrument from Roche Diagnostics, as previously described [30]. Serum levels of selected proteins (i.e. MPO, HNP1-3, BD-1, and BD-2) were measured by enzyme immunoassays (EIA) as previously described [30, 31].

### Global microarray-based transcriptome analysis

Total RNA (200 ng) was reverse transcribed, amplified and biotin-labelled using the Illumina Total Prep RNA amplification kit (Ambion, Huntingdon, UK). Biotin-labelled complementary RNA (750 ng) was hybridized to Illumina Human HT-12 v4 Bead Chips (Illumina, San Diego, CA, USA), according to the manufacturer’s instructions. These chips contain more than 47,000 probes, selected primarily from the NCBI Refseq database (release 38). Following hybridization, the bead chips were washed and stained with Streptavidin-Cy3 (Thermo Fisher Scientific, Waltham, MA, USA). Fluorescent signal detection was performed by the iScan Reader (Illumina). The resulting images were processed by Genome Studio Software v2009.1 (Illumina), and tables with sample annotation and expression values (GeneSpring-format) and control values were exported.

### Data processing and statistical analyses

Data processing and statistical analyses were performed in R [32] using the packages Limma [33] (background correction as described in [34], quantile normalization, log2-transformation, adjustment of technical batch effects and statistics), Lumi [35] (outlier detection), illuminaHumanv4.db [36] (update of probe annotation), and sva [37] (identification of surrogate variables to remove artefacts caused by technical batch effects). Additional details of the microarray data processing are given in supplementary methods and results, Fig. S1 and Table S1.

In total, 1891 microarray-based gene expression samples passed the initial technical quality control and were included for quantile normalization and batch adjustment (Fig. S1). For the downstream analyses, we merged the gene expression data set with the TOP clinical data and filtered on some additional exclusion criteria: (1) blood-sampling time was restricted to between 7 and 11 am to minimize the time-of-day effect on white blood cells, (2) non-fasting status, and (3) C-reactive protein (CRP) > 10. After quality control and post-processing steps the final set had 721 samples: SCZ (*N* = 329), BD (*N* = 203), and HC (*N* = 189).

We used the Empirical Bayes approach of the ‘limma’ R package [38] to estimate the effect of the diagnostic groups on the gene expression levels by fitting a linear model while correcting for technical variables (plate, biotin, run), age and sex. Estimated sample variance is shrunk by the Empirical Bayes method while variance between groups is handled by the function arrayWeights(). Correction for multiple testing was performed using the Benjamini and Hochberg False Discovery Rate (FDR < 0.05). Similar to the differential gene expression analysis between diagnostic groups, the effect of tobacco smoking was assessed by comparing smokers and non-smokers within the patient groups and adjusting for technical variables (plate, biotin, run), age and sex. For genes with multiple probes, we only report the one with the highest log2 fold change. Probes with no matching Gene symbol and Entrez ID were not included in the final tables of differentially expressed genes (DEGs).

The overlap between DEGs (*p*-value < 0.05) in SCZ vs HC and BD vs HC was calculated with the GeneOverlap package in R [39]. Hypergeometric *p*-value was calculated from a total of 21,215 unique gene entities present on the Illumina HumanHT-12 v4 Expression BeadChip (Bioconductor package: illuminaHuman4.db v.1.26.0).

We generated gene expression correlation matrices of the top 10 differentially up- and downregulated genes and selected serum markers (i.e., cholesterol, triglyceride, MPO, HNP13, BD1, and BD2). The correlation matrices were visualized with the corrplot R package. Gene distribution and sex differences were visualized with violin plots based on diagnosis.

Post hoc analyses were performed for SCZ and BD DEGs and selected variables (sex, age, BMI, CRP, serum markers (i.e., cholesterol, triglyceride, MPO, HNP13, BD1, and BD2), and PANSS score. For the PANSS score, we included patients that had their PANSS interview and blood sampling within a timeframe of maximally 30 days (*N* = 260 SCZ and 155 BD). For continuous variables, we used the corr.test function in the psych R package to calculate the relationship between DEGs and covariates using Pearson’s correlation coefficient (*r*) and Spearman’s rho. The single effect of sex on DEGs was estimated by the one-way ANOVA model. Antipsychotic use was grouped by type of drug for groups that were of adequate size to be compared (i.e., olanzapine, quetiapine, aripiprazole, and antipsychotic non-users). Differences in gene expression between antipsychotic groups were calculated using the one-way ANCOVA model with sex, age, and diagnosis as covariates with Emmeans for the post hoc pairwise comparison between groups. For groups that did not meet the assumptions of the ANCOVA model (assessed with Shapiro test and Levene test), statistical analyses were performed with Welch’s anova.

### Functional profiling of differentially expressed genes

To identify biological pathways that were overrepresented among the DEGs, we used gene set enrichment analysis (GSEA, https://www.gsea-msigdb.org/) with preranked gene list (rank = fold change * −log10(adj.*p*-value)) and the gene ontology dataset for biological pathways (c5.go.bp).

## Results

### Demographic data of the study participants

The study included 329 SCZ, 203 BD, and 189 HC after quality control. There were some significant between-group differences in the demographic data (Table 1). The BD group had fewer males compared to the other two groups, and there was a lower mean age in the SCZ group.

**Table 1:** Demographic and clinical characteristics of study participants.

	Schizophrenia	Bipolar disorder	Healthy controls	*p*-value
**Participants,** ***n***	329	203	189	
**Age (years), mean (SD)**	30.4 (9.6)*	33.9 (11.7)	32.6 (8.7)	<0.008
**Sex,** ***n*** **(%):**				
Male	211 (64.1)	82 (40.4)*	115 (60.8)	<0.001
Female	118 (35.9)	121 (59.6)	74 (39.2)	
**Ethnicity,** ***n*** **(%):**				
European	267 (81.2)*	181 (89.2)*	187 (98.9)*	<0.01
Non-European	62 (18.8)	22 (10.8)	2 (1.1)	
**Diagnosis of schizophrenia,** ***n*** **(%):**				
Schizophrenia	238 (72.3)	NA	NA	
Schizoaffective	61 (18.5)			
Schizophreniform	30 (9.1)			
**Diagnosis of bipolar disorder,** ***n*** **(%):**				
Bipolar I	NA	147 (72.4)	NA	
Bipolar II		44 (21.7)		
Bipolar NOS		12 (5.9)		
**Age of onset psychosis (years), mean (SD)**	23.7 (7.9)	26.8 (9.5)	NA	0.001
Based on data from *n* (%) subjects:	312 (94.8)	129 (63.5)		
**PANSS, mean (SD)**	64.1 (16.9)	45.1 (9.7)	NA	<0.001
**Current antipsychotic users,** ***n*** **(%)**	281 (85.4)	112 (55.2)	NA	0.002
Aripiprazole	46	12		
Clozapine	10	0		
Olanzapine	93	53		
Paliperidone	10	0		
Quetiapine	61	37		
Risperidone	35	4		
Other^a)^	26	6		
Non-users	48	91		
**Current lithium users,** ***n*** **(%)**	10 (3.0)	37 (18.2)	NA	<0.001
**Tobacco,** ***n*** **(%):**				
Smokers (daily use)	164 (49.8)	95 (46.8)	23 (12.2)*	<0.02
Non-smokers	160 (48.6)	106 (52.2)	50 (26.5)	
NA	5 (1.5)	2 (1.0)	116 (61.4)	
**BMI (kg/m**^**2**^**), mean (SD)**	26.6 (5.1)*	25.0 (4.5)	24.9 (3.9)	˂0.032
Based on data from n (%) subjects:	175 (53.2)	93 (45.8)	35 (18.5)	
**CRP (mg/L), mean (SD)**	2.1 (2.1)	1.9 (1.9)	1.4 (1.6)*	<0.008

Statistical difference between groups was calculated by diagnosis vs HC, except for measurements that were only available/relevant for patients, i.e., age of onset, medication, and PANSS. Statistical models used were Chi square and pairwise t test.

*BMI* body mass index, *n* number of subjects, *NA* not applicable, *NOS* not otherwise specified, *SD* standard deviation.

* Indicates which group is significantly different from the other two.

^a^Other antipsychotics (less than 10 users in both patient groups): Amisulpride, Chlorprothixene, Flupentixol, Perphenazine, Sertindole, Ziprasidone, Zuklopentixol.

As expected, the SCZ group had more severe psychosis symptoms compared to the BD group (PANSS total median (min-max) of 64 (31–144) for SCZ and 43.5 (30–85) for BD), and SCZ patients also had earlier age of onset and longer duration of psychosis compared to BD patients.

More SCZ patients (85%) were on antipsychotic treatment compared to BD patients (55%). Most of the antipsychotic users were on olanzapine (33% SCZ, 47% BD), quetiapine (22% SCZ, 33% BD), aripiprazole (16% SCZ, 11% BD) or risperidone (12% SCZ, 4% BD).

Compared to the HC, patients had slightly higher CRP level also after excluding participants with CRP above 10. Data on BMI and tobacco smoking was mainly available for the patients.

### Shared gene expression signatures for SCZ and BD point to changes in neutrophil granulocytes

**Fig. 1:**
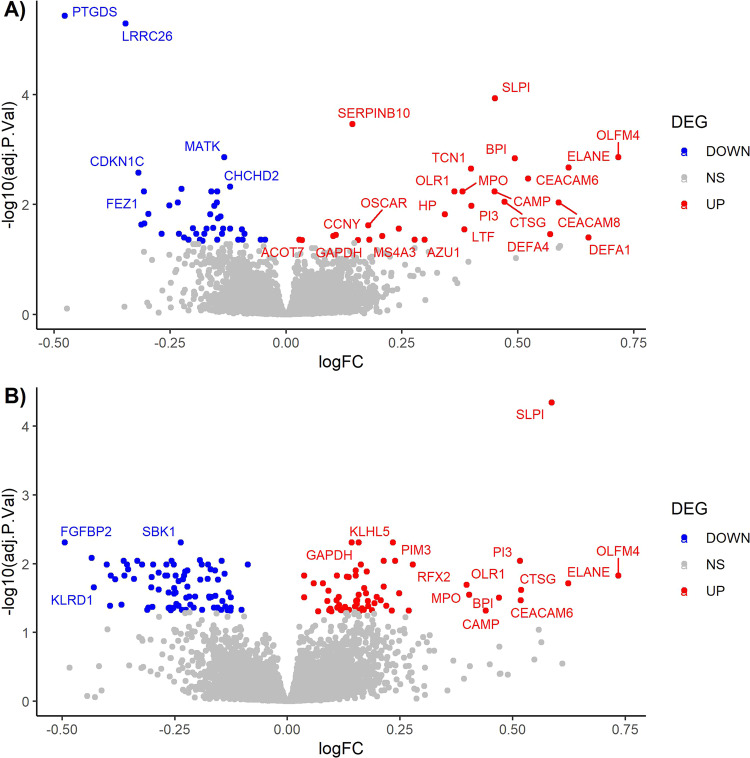
Volcano plot of the differentially expressed genes. **A** Schizophrenia vs healthy controls. **B** Bipolar disorder vs healthy controls. NS = not significant.

The microarray-based global transcriptome analysis in peripheral blood identified 65 genes differentially expressed in SCZ and 125 in BD, compared to HC (age and sex adjusted, FDR-adjusted *p*-value < 0.05, Fig. 1, Table 2, and Table S3). The overall expression changes were markedly similar for the two disorders, with almost equal ratio of up- and downregulated genes (27 up/38 down in SCZ, 58 up/67 down in BD), no transcripts with absolute FC > 1.70 (Table 2) and a significant overlap in DEGs (20 genes, hypergeometric overlap: *p*-value = 1.4E−48).

**Table 2:** Overview of differentially expressed genes after adjusting for age and sex.

Gene symbol	Gene name	Entrez ID	log2FC	FC	adj.*p*-value
**(A) Differentially expressed genes in SCZ vs HC**					
*Top 20 upregulated genes in schizophrenia*					
OLFM4	Olfactomedin 4	10562	0.72	1.64	0.001
DEFA1	Defensin alpha 1	1667	0.65	1.57	0.040
ELANE	Elastase, neutrophil expressed	1991	0.61	1.53	0.002
CEACAM8	Carcinoembryonic antigen related cell adhesion molecule 8	1088	0.59	1.50	0.009
DEFA4	Defensin alpha 4	1669	0.57	1.48	0.035
CEACAM6	Carcinoembryonic antigen related cell adhesion molecule 6	4680	0.52	1.44	0.003
BPI	Bactericidal permeability increasing protein	671	0.49	1.41	0.001
CTSG	Cathepsin G	1511	0.47	1.39	0.009
SLPI	Secretory leukocyte peptidase inhibitor	6590	0.45	1.37	1.16E−04
CAMP	Cathelicidin Antimicrobial Peptide	820	0.45	1.37	0.006
PI3	Peptidase inhibitor 3	5266	0.40	1.32	0.010
TCN1	Transcobalamin 1	6947	0.40	1.32	0.002
LTF	Lactotransferrin	4057	0.38	1.31	0.028
MPO	Myeloperoxidase	4353	0.38	1.30	0.006
OLR1	Oxidized low density lipoprotein receptor 1	4973	0.36	1.29	0.006
HP	Haptoglobin	3240	0.34	1.27	0.015
AZU1	Azurocidin 1	566	0.30	1.23	0.044
MS4A3	Membrane Spanning 4-Domains A3	932	0.28	1.21	0.044
CYP1B1	Cytochrome P450 Family 1 Subfamily B Member 1	1545	0.24	1.18	0.028
FBN2	Fibrillin 2	2201	0.21	1.15	0.037
*Top 20 downregulated genes in schizophrenia*					
PTGDS	Prostaglandin D2 Synthase	5730	−0.48	−1.39	3.66E−06
LRRC26	Leucine Rich Repeat Containing 26	389816	−0.35	−1.27	5.09E−06
CDKN1C	Cyclin dependent kinase inhibitor 1C	1028	−0.32	−1.25	0.003
CD160	CD160 Molecule	11126	−0.31	−1.24	0.023
FEZ1	Fasciculation And Elongation Protein Zeta 1	9638	−0.31	−1.24	0.006
AKR1C3	Aldo-Keto Reductase Family 1 Member C3	8644	−0.31	−1.24	0.022
SH2D1B	SH2 Domain Containing 1B	117157	−0.30	−1.23	0.015
CLIC3	Chloride Intracellular Channel 3	9022	−0.27	−1.20	0.034
LYPD2	LY6/PLAUR Domain Containing 2	137797	−0.25	−1.19	0.010
TAGLN	Transgelin	6876	−0.23	−1.18	0.009
PTGDR	Prostaglandin D2 Receptor	5729	−0.23	−1.17	0.034
PACSIN1	Protein Kinase C And Casein Kinase Substrate In Neurons 1	29993	−0.23	−1.17	0.005
TNFRSF21	TNF Receptor Superfamily Member 21	27242	−0.22	−1.16	0.040
MS4A7	Membrane Spanning 4-Domains A7	58475	−0.20	−1.15	0.027
AKR1C4	Aldo-Keto Reductase Family 1 Member C4	1109	−0.19	−1.14	0.034
PLEKHF1	Pleckstrin Homology And FYVE Domain Containing 1	79156	−0.19	−1.14	0.044
KLRB1	Killer Cell Lectin Like Receptor B1	3820	−0.18	−1.13	0.046
CEP78	Centrosomal Protein 78	84131	−0.18	−1.13	0.034
MYOF	Myoferlin	26509	−0.17	−1.13	0.028
DUSP5	Dual Specificity Phosphatase 5	1847	−0.16	−1.12	0.015
**(B) Differentially expressed genes in BD vs HC**					
*Top 20 upregulated genes in bipolar disorder*					
OLFM4	Olfactomedin 4	10562	0.73	1.66	0.015
ELANE	Elastase, neutrophil expressed	1991	0.62	1.54	0.019
SLPI	Secretory leukocyte peptidase inhibitor	6590	0.59	1.50	4.51E−05
CTSG	Cathepsin G	1511	0.52	1.43	0.024
CEACAM6	Carcinoembryonic antigen related cell adhesion molecule 6	4680	0.52	1.43	0.034
PI3	Peptidase inhibitor 3	5266	0.52	1.43	0.009
BPI	Bactericidal permeability increasing protein	671	0.47	1.38	0.031
CAMP	Cathelicidin Antimicrobial Peptide	820	0.44	1.36	0.048
MPO	Myeloperoxidase	4353	0.40	1.32	0.028
OLR1	Oxidized low density lipoprotein receptor 1	4973	0.40	1.32	0.020
RFX2	Regulatory Factor X2	5990	0.28	1.21	0.010
ADGRE1	Adhesion G Protein-Coupled Receptor E1	2015	0.27	1.21	0.048
H1-0	H1.0 Linker Histone	3005	0.25	1.19	0.027
OSCAR	Osteoclast Associated Ig-Like Receptor	126014	0.24	1.18	0.009
PIM3	Pim-3 Proto-Oncogene, Serine/Threonine Kinase	415116	0.23	1.18	0.005
SLC2A5	Solute Carrier Family 2 Member 5	6518	0.23	1.17	0.048
FAR2	Fatty Acyl-CoA Reductase 2	55711	0.22	1.16	0.041
GAS7	Growth Arrest Specific 7	8522	0.21	1.16	0.009
FES	FES Proto-Oncogene, Tyrosine Kinase	2242	0.21	1.15	0.034
ZNF438	Zinc Finger Protein 438	220929	0.20	1.15	0.031
*Top 20 downregulated genes in bipolar disorder*					
FGFBP2	Fibroblast growth factor binding protein 2	83888	−0.49	−1.41	0.005
ADGRG1	Adhesion G protein-coupled receptor G1	9289	−0.43	−1.35	0.008
KLRD1	Killer Cell Lectin Like Receptor D1	3824	−0.43	−1.35	0.022
GNLY	Granulysin	10578	−0.39	−1.31	0.041
PTGDS	Prostaglandin D2 Synthase	5730	−0.39	−1.31	0.015
CD160	CD160 Molecule	11126	−0.38	−1.30	0.017
ITGB1BP1	Integrin Subunit Beta 1 Binding Protein 1	9270	−0.37	−1.29	0.039
HOPX	HOP Homeobox	84525	−0.36	−1.29	0.009
CDKN1C	Cyclin dependent kinase inhibitor 1C	1028	−0.36	−1.28	0.010
GZMB	Granzyme B	3002	−0.35	−1.28	0.012
S1PR5	Sphingosine-1-Phosphate Receptor 5	53637	−0.34	−1.27	0.017
CCL4	C-C Motif Chemokine Ligand 4	6351	−0.33	−1.26	0.009
FASLG	Fas Ligand	356	−0.32	−1.25	0.010
PRSS23	Serine Protease 23	11098	−0.31	−1.24	0.047
CLIC3	Chloride Intracellular Channel 3	9022	−0.31	−1.24	0.043
CCL4L2	C-C Motif Chemokine Ligand 4 Like 2	9560	−0.30	−1.23	0.022
PRF1	Perforin 1	5551	−0.30	−1.23	0.016
IFNG	Interferon Gamma	3458	−0.30	−1.23	0.042
NKG7	Natural Killer Cell Granule Protein 7	4818	−0.30	−1.23	0.010
SAMD3	Sterile Alpha Motif Domain Containing 3	154075	−0.29	−1.22	0.013

*SCZ* schizophrenia, *BD* bipolar disorder, *HC* healthy control, *FC* fold change.

Downregulated genes are calculated as −1/FC. Adj.*p*-value is Bonferroni-adjusted *p*-value.

Many of the upregulated genes (16 in SCZ and 9 in BD, e.g., *ELANE, CEACAM6, BPI, CTSG*, and *MPO*) encode proteins that are enriched in neutrophils (www.proteinatlas.org). The downregulated genes were more diverse between the two disorders, but many of the shared downregulated genes (e.g., *PTGDS, CD160, CLIC3*, and *PTGDR*) are enriched in dendritic cells (DC), natural killer cells (NK) and T-cells (www.proteinatlas.org). In support of this, patients had lower proportions of resting NK cells and activated CD4 memory T-cells as estimated by CIBERSORT analysis (Supplementary methods and results, Fig. S2). Interestingly, none of the genes were differentially expressed between SCZ and BD after correcting for multiple testing (Table S3).

The differential expression analysis was performed with adjustment for sex and age. In addition, we performed post hoc analysis to estimate the single effect of sex on the DEGs. The expression level of the neutrophil-related genes was significantly higher in males than females for all groups (SCZ, BD, and HC, *p*-value < 0.05; Table S4). Figure S3 shows that the sex difference in the expression of *ELANE* and *CEACAM6* was observed in both patients and controls, but within different ranges of expression values, and with large interindividual differences in all phenotype groups. In contrast, two elastase inhibitors, *PI3* and *SLPI*, did not show a similar sex difference, and there were less interindividual differences in their expression levels (Fig. S3 and Table S4).

### Gene annotation and correlation analysis indicate innate immune function and association with lipid changes

We used GSEA to identify which biological pathways the significant DEGs were associated with (Table 3). The upregulated genes in both SCZ and BD were positively associated with defence response and antibacterial activity. In contrast, the GSEA did not return any significant hits (FDR *q*-value < 0.05) for negatively regulated biological pathways for SCZ or BD.

**Table 3:** Biological pathway analysis of differentially expressed genes.

NAME	SIZE	ES	NES	NOM *p*-val	FDR *q*-val	FWER *p*-val
**(A) Enriched positively regulated pathways in SCZ (FDR** **<** **0.05)**						
Killing by host of symbiont cells	27	0.93	1.80	<0.001	<0.001	<0.001
Defense response to fungus	49	0.90	1.77	<0.001	5.0E−4	0.001
Antibacterial humoral response	53	0.89	1.75	<0.001	0.001	0.001
Organ or tissue specific immune response	38	0.89	1.74	<0.001	0.001	0.001
Innate immune response in mucosa	24	0.90	1.73	<0.001	0.001	0.001
Response to fungus	60	0.87	1.72	<0.001	0.001	0.001
Defense response to gram negative bacterium	80	0.86	1.71	<0.001	0.001	0.001
Antimicrobial humoral response	114	0.83	1.68	<0.001	0.001	0.134
Antimicrobial humoral immune response mediated by antimicrobial peptide	74	0.84	1.68	<0.001	0.001	0.159
Modulation of process of another organism	22	0.88	1.68	<0.001	0.001	0.166
Negative regulation of interleukin 8 production	30	0.86	1.67	<0.001	0.001	0.227
**(B) Enriched positively regulated pathways in BD (FDR** **<** **0.05)**						
Modulation of process of another organism	22	0.88	1.67	<0.001	0.001	0.105
Antibacterial humoral response	53	0.85	1.66	<0.001	0.001	0.151
Response to fungus	60	0.83	1.64	<0.001	0.001	0.208
Nucleoside catabolic process	26	0.85	1.64	<0.001	0.001	0.261

Significantly enriched gene ontology biological pathways (GOBP) returned from GSEA on preranked gene list where rank = FC*(−log10(adj.*p*-value)).

*SIZE* Number of genes from the expression data set that was found in the gene set. *ES* Enrichment score (the degree to which a gene set is overrepresented), *NES* Normalized enrichment score, *NOM p-val* Nominal *P* value, *FDR* False discovery rate, *FWER* Familywise-error rate.

**Fig. 2:**
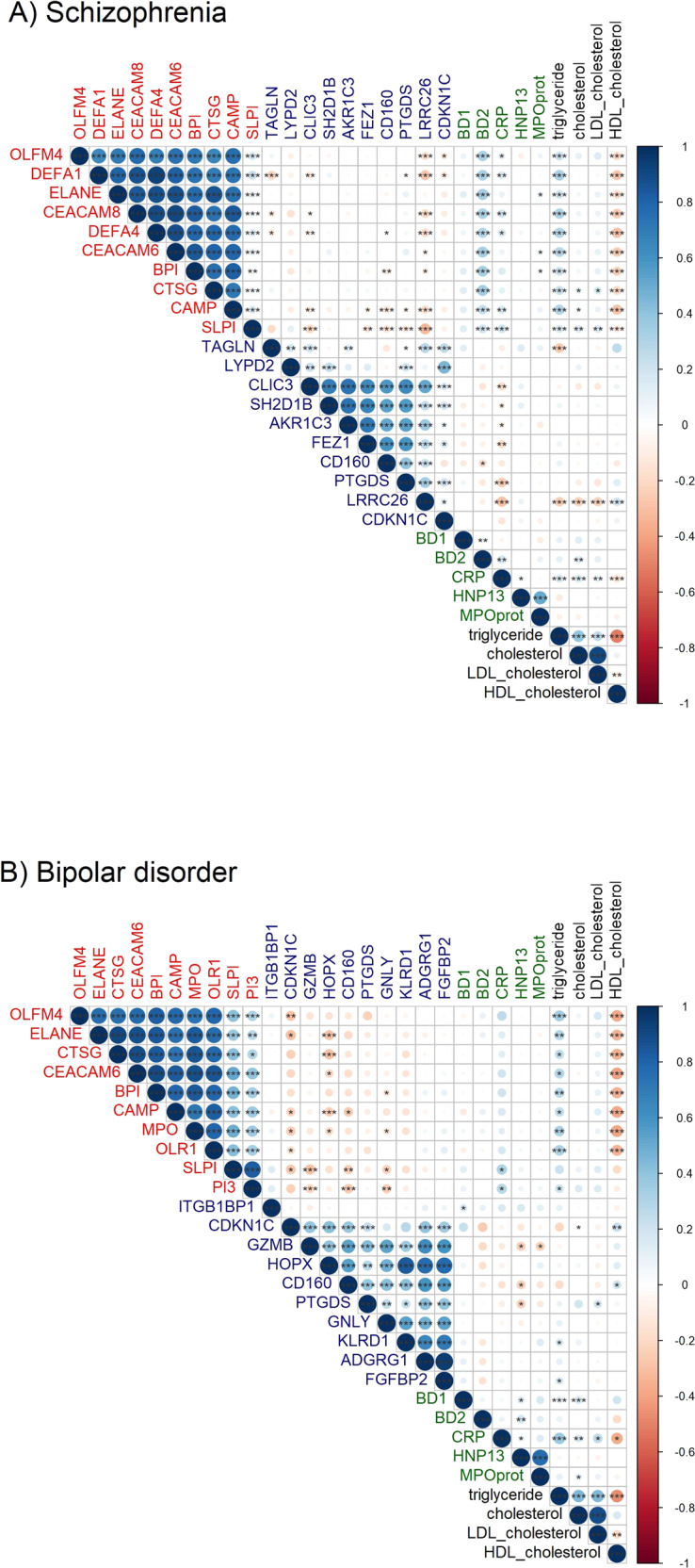
Co-expression matrix of top 10 differentially expressed genes (up- and downregulated) and selected blood markers. **A** Schizophrenia and (**B**) Bipolar disorder. Upregulated genes are written in red, downregulated genes in blue, serum proteins in green, and serum lipids in black. Corresponding to the strength of Pearson correlation, a positive co-expression is indicated in a gradient of blue and a negative co-expression is indicated in a gradient of red. Genes are annotated with their gene symbol. Serum protein annotation: BD1 beta defensin 1, BD2 beta defensin 2, CRP C-reactive protein, HNP13 human neutrophil peptide 1 and 3, MPOprot MPO protein (to differentiate it from the gene with the same name). Significance level is indicated with *p-value < 0.05, **p-value < 0.01 and ***p-value =≤ 0.001.

We examined the gene-to-gene correlation matrices and found a high degree of correlation among the top-ranked genes, displayed as two main clusters of the up- and downregulated genes, respectively (Fig. 2). In SCZ, but not in BD, many of the common upregulated genes were also positively statistically significantly associated with the level of soluble beta-defensin 2 (BD2) protein. We did not find any association between the expression of neutrophil-related genes and the blood levels of soluble neutrophil granule proteins (neutrophil defensin 1 (HNP13) and myeloperoxidase (MPO)) in any of the two disorders.

Interestingly, when we included data on serum markers in the correlation analyses (Fig. 2, Table S5), we found that the upregulated genes were positively correlated with triglycerides (Pearson’s *r* ≤ 0.35, *p*-value < 0.02) and negatively correlated with HDL-cholesterol level (Pearson’s *r* ≥ −0.32, *p*-value < 0.01) in both disorders. Only one gene was correlated with cholesterol in both disorders (*TNFRSF21*, Pearson’s *r* = ≥−0.20, *p*-value < 0.03) and one with LDL-cholesterol (*LRRC26*, Pearson’s *r* = ≥−0.21, *p*-value < 0.04).

Furthermore, we found that BMI correlated significantly with the expression of many of the upregulated neutrophil-related genes in both disorders (Pearson’s *r* ≤ 0.29, p-value < 0.04), but missing data may have reduced the statistical significance of some of these associations. BMI showed the strongest negative correlation with the gene *LRRC26* in both SCZ (Pearson’s *r* = −0.39, *p*-value = 1.38E−07) and BD (Pearson’s *r* = −0.34, *p*-value = 0.0015).

With respect to age, the most consistent gene relationships in both disorders were observed for the expression of *KLRB1* with negative correlation (SCZ: Pearson’s *r* = −0.22, *p*-value = 4.3E−05, BD: Pearson’s *r* = −0.27, *p*-value = 0.0002) and *CDKN1C* with positive correlation (SCZ: Pearson’s *r* = 0.19, *p*-value = 0.0005, BD: Pearson’s *r* = 0.18, *p*-value = 0.01).

In both disorders, the inflammation marker CRP showed the strongest positive association with the genes *HP, SLPI*, and *NCF4* (SCZ: Pearson’s *r* ≤ 0.37, *p*-value < 4.73E-6, BD: Pearson’s *r* ≤ 0.32, *p*-value < 3.01E−05) and a negative association with the genes *TNFRSF21, LRRC26*, and *PACSIN1*, (SCZ: Pearson’s *r* ≥ −0.31, *p*-value < 6.03E−07, BD: Pearson’s *r* ≥ −0.24, *p*-value ˂ 0.0014). Regarding the neutrophil-related genes, none of them were significantly associated with CRP in BD, while some of them (*CAMP, CEACAM8, DEFA1, DEFA4, LTF* and *OLFM4*) were very weakly correlated with CRP in SCZ (Pearson’s *r* ≤ 0.18, *p*-values < 0.04).

### Tobacco smoking markedly influence the gene expression pattern

Tobacco smoking is known to influence gene expression in blood cells [40], and to assess the effect of tobacco smoking, we analyzed the gene expression between smokers and non-smokers within the patient groups (Table S6). We found a large effect of smoking between the groups, with 177 significant DEGs in SCZ and BD smokers compared to non-smokers. Sixteen of the “tobacco smoking” DEGs were overlapping with the DEGs in SCZ vs HC (hypergeometric overlap: *p*-value = 1.2E−19). Most of these genes were downregulated and only one was upregulated (i.e., *ACOT7*). For BD, the overlap between BD vs HC DEGs and smoking DEGs were 31 genes (hypergeometric overlap: *p*-value = 2.2E−37), of which all were downregulated in BD. There was a significant difference in the number of DEGs that survived correction for multiple testing (adjusted *p*-value < 0.05) when analyzing SCZ (149 DEGs) and BD (1 DEG, i.e., *LRRN3*) separately for smokers vs non-smokers (Table S6).

### Association of gene expression with symptoms and treatment

To investigate if the differential expression pattern was linked to symptoms, we performed correlation analysis of age of onset (aoo) and PANSS score (total, positive, negative, and general, Table S7). In both disorders, we found a weak negative correlation between aoo and *KLRB1* (Pearson’s *r* ≥ −0.24, *p*-value < 0.01). In BD, aoo also correlated positively with some neutrophil-related genes (i.e., *SLPI, MS4A3, TCN1, PI3*; Pearson’s *r* ≤ 0.24, *p*-value < 0.02). Interestingly, there was a weak correlation between the elastase inhibitor *PI3* and PANSS negative and PANSS positive in both disorders (Pearson’s *r* ≤ 0.21, *p*-value < 0.04). *PI3* also correlated with all PANSS scores in SCZ with the strongest significance in PANSS total and PANSS positive. Many of the neutrophil-related genes were weakly correlated with PANSS in BD (Pearson’s *r* ≤ 0.23, *p*-value < 0.05), but not in SCZ. In both disorders, we found a negative correlation between *HDDC2* and PANSS total and PANSS positive (Pearson’s *r* ≥ −0.24, *p*-value < 0.03), as well as a negative correlation between *RHOC* and PANSS total (Pearson’s *r* ≥ −0.18, *p*-value < 0.03).

To determine if the gene expression was influenced by medication, we compared the expression pattern of different treatment groups. We identified four main treatment groups when examining the main antipsychotics used by the study participants: olanzapine users (*N* = 93 SCZ, 53 BD), quetiapine users (*N* = 61 SCZ, 37 BD), aripiprazole users (*N* = 46 SCZ, 12 BD), and antipsychotic non-users (*N* = 48 SCZ, 91 BD). There was a significant higher expression level of the genes *PTGDS*, *CLIC*, and *GZMB* in aripiprazole users compared to all the other groups (adj.*p*-value < 0.03, Fig. S4A–C and Table S2). Quetiapine users had a lower level of *TOGARAM2* compared to the other groups (adj.*p*-value < 0.03, Fig.S4D and Table S2). The group of antipsychotic non-users had significant higher expression of *SIDT2* compared to the antipsychotic-users (adj.*p*-value = 0.02, Fig. S4E and Table S2).

## Discussion

### SCZ and BD have elevated level of immature neutrophil-related genes

Our main finding is that SCZ and BD display a similar gene expression signature in peripheral blood, that strongly reflects innate immune responses and changes in neutrophil granulocytes.

The top upregulated DEGs in SCZ and BD, which encode neutrophil granule proteins, had a highly correlated expression pattern. Previous studies have identified some of the same innate immunity genes in SCZ, including genes encoding neutrophil defensins and protease inhibitors [21, 27, 28, 41, 42]. Neutrophil granulocytes are important mediators of inflammatory responses associated with infections and tissue injury. They provide rapid clearance of pathogens, but they also play an active role in several non-infectious conditions, such as autoimmune diseases and cancer [43–45]. To fulfil their broad range of functions, neutrophils produce a large number of effector molecules, including proteases (e.g. neutrophil elastase, CTSG), antimicrobial peptides (e.g. neutrophil defensin 1)), cytokines and reactive oxygen intermediates (catalyzed by the peroxidase MPO), and they release extracellular traps [44, 46].

The upregulated genes with highly correlated expression are transcribed by developing granulocytes in the bone marrow (promyelocytes and myelocytes) [47]. The increased expression of *DEFA1* and *MPO* did not correlate with the levels of the corresponding serum proteins (neutrophil defensin 1 and MPO). These proteins are normally stored in neutrophil granules. Mature neutrophils contain different granule subsets that are characterized by distinct protein contents [48]. Each granule subset is produced at a specific stage of terminal neutrophil differentiation and is only filled with those proteins that are synthesized at the time when the granule subsets are formed. The granules will therefore be representative for the cell type in which they were formed [49]. Most of the upregulated genes in SCZ and BD codify for mRNAs that are primarily transcribed during the promyelocytic (*DEFA1, ELANE, CEACAM6, CTSG, MPO, MS4A3*) and myelocytic (*BPI, OLFM4, LTF, HP, LCN2, CAPM, CEACAM8*) stages of neutrophil differentiation, suggesting that the immature neutrophils described here represent a heterogenous population composed primarily of promyelocytes and myelocytes [49]. The release of developing neutrophils into the blood is a feature of stress-induced myelopoiesis in response to immunological triggers [50]. This may indicate that the gene expression signature in peripheral blood of SCZ and BD reflects an increased proportion of immature neutrophils, rather than an increase in cellular gene expression, per se.

### Immune findings in SCZ and BD

Our data indicate changes in innate immune responses and adds novel information to the role of the immune system in SCZ and BD patients. Several epidemiological studies have shown that both maternal infections and infections prior to diagnosis are associated with increased risk of later diagnosis of SCZ and BD [51–55] and that patients with SCZ have an increased risk of co-morbid autoimmune disorders [56–60]. Other studies have reported that the level of proinflammatory cytokines in peripheral blood is increased in patients with SCZ and BD, including interleukin 6 (IL-6) and tumour necrosis factor-alpha (TNF-α) [61–64]. These proinflammatory cytokines regulate the production of CRP, and increased serum levels of CRP has been reported in SCZ and BD [65, 66], and was also observed for SCZ and BD patients in our study.

The neutrophil transcriptome signature observed in our study also adds new knowledge to the role of neutrophils in psychiatric diseases. Neutrophil count in peripheral blood has been associated with severity of psychosis (i.e. higher total PANSS score) and reduced MRI-determined grey matter volume in the brain [67]. Moreover, it has been reported that FEP and SCZ have higher neutrophil-lymphocyte ratio (NLR) compared to HC, with reduction after six weeks of treatment, suggesting that the NLR count may be a useful measure for disease severity and treatment response [68]. Interestingly, immature granulocytes are associated with early-stage infections and have been shown to be better markers than CRP and IL-6 for systemic inflammatory response syndrome [69].

We found that *PI3* correlated positively with PANSS negative and PANSS positive in both disorders. In BD, we also identified an association between immature neutrophil genes and PANSS. Interestingly, genes of immature neutrophils were also differentially expressed in a study comparing best-responders and worst-responders after three months of antipsychotic treatment [70] as well as in patients with antipsychotic-induced weight gain [42].

### The immature neutrophil expression signal is associated with lipid changes in SCZ and BD

The link between antipsychotic treatment and weight gain is well established and the drugs are in varying degree associated with metabolic changes, including increased level of triglyceride and lower level of HDL-cholesterol [71]. Our post-hoc analysis demonstrated that the immature neutrophil expression profile in SCZ and BD was positively associated with the serum level of triglyceride and negatively associated with the HDL-cholesterol level. A similar correlation was reported for the expression of the genes *ELANE* and *MPO* with triglyceride level and BMI in overweight and obese individuals [72]. In patients with SCZ and BD, this may suggest that immature neutrophils are changed in response to antipsychotic-related side effects, such as weight gain and dyslipidaemia. However, we did not find any difference in the expression of the immature neutrophil-related genes between antipsychotic users and non-users. A possible explanation may be the presence of metabolic syndrome in many drug-naïve patients [73].

Many of the upregulated immature neutrophil genes (e.g., *OLFM4*, *DEFA1, ELANE, CEACAM6, CEACAM8, CTSG*) have been found to be differentially expressed in persons with obesity and type 2 diabetes [74, 75], both common comorbidities with psychotic disorders. Interestingly, a causal role for the protein neutrophil elastase in insulin resistance and adipose tissue inflammation has been demonstrated in mice [76]. Recent evidence indicate that in response to adipocyte stress, neutrophils infiltrate adipose tissue and initiate inflammation through signalling to macrophages and other immune cells (reviewed by [77]).

Two of the downregulated genes, TNF receptor superfamily member 21 (*TNFRSF21*) and Leucine rich repeat containing 26 (*LRRC26*), were negatively correlated with triglyceride and LDL-cholesterol. *LRRC26* was also negatively correlated with the immature neutrophil genes. *TNFRSF21* is a member of the TNF receptor family, and knockout studies have demonstrated a regulatory role for this receptor in adaptive immunity [78]. *LRRC26* encodes the gamma 1 auxiliary subunit of the large-conductance, Ca2+- and voltage-activated K+ (BK) channel [79]. The γ1 subunit is found on secretory, non-excitable cells and downregulates the BK channel from high-voltage to low-voltage activation [80]. Downregulation of *LRRC26* expression can be mediated by IFN-γ, TGF-β1 or promotor methylation [80]. *TNFRSF21* and *LRRC26* gene expression was also associated with CRP and BMI in our study.

### Downregulated genes in SCZ and BD may reflect tobacco smoking

Tobacco smoking is more frequent among individuals with SCZ and BD compared to the general population [81], and smokers with psychiatric disorders also have high prevalence of nicotine dependence and high cigarette consumption [82]. A genetic link between SCZ and nicotine dependence has been reported [83] and tobacco smoking leaves a footprint on DNA methylation [84] and gene expression [40] profiles. To determine if some of the DEGs were confounded by smoking, we compared smoking and non-smoking SCZ and BD patients. Many of the top listed genes are known smoking-related genes like *LRRN3, SASH1, PID1*, and *S1PR5* [40, 85]. We found a large overlap between the DEGs identified between smoking and non-smoking patients in our dataset and the top-ranked downregulated genes (e.g., *CDKN1C, FEZ1, CD160, KLRB1, FGFBP2*, and *ADGRG1*) in patients versus controls. A similar gene profile was also characteristic for 743 smokers compared to 1686 never-smokers in a study with the aim to identify molecular pathways involved in smoking [85]. In addition, the authors identified a time-dependent normalization of smoking-related genes up to 5 years after quitting smoking. The large difference in DEGs when analyzing SCZ and BD separately, was unexpected and may be a power-issue or the dose-dependent effect of tobacco smoking [85].

### Blood versus brain transcriptomics

Transcriptomic studies from SCZ show a mixed picture [86], reflecting the heterogeneity and complexity of the disease combined with differences in study designs, cohorts, and tissues that have been studied. Brain transcriptomics are more likely to reflect pathology, while peripheral blood samples, on the other hand, may reflect disease-related symptoms or treatment effects [87, 88]. However, immune dysregulations have been identified in both brain and blood transcriptomics [18, 19, 21–23, 28]. Studies on post-mortem brain tissue have verified some of the genetic findings in SCZ and BD, such as changes in complement pathway genes (i.e., *C4* and *HLA-DPA1*) encoded by the major histocompatibility complex region [18, 89]. Many studies of the blood transcriptomics in SCZ and BD have reported dysregulation of the innate immune system [21, 23, 28], but up until now, the immature neutrophils cells have not been emphasized.

### Limitations of the study

Our study applied a microarray-based global gene expression platform. The main disadvantage of this technology compared to RNA-sequencing, is potential non-specific hybridization that may induce false positive results. However, the strong gene-to-gene co-expression clusters, with 16 (SCZ) and 9 (BD) genes encoded by the same cell type among the most upregulated genes strongly suggest that this is not a by-chance finding. In addition, similar findings in other cohorts support our conclusion of an elevated level of immature neutrophil genes in SCZ and BD [21, 28, 87].

White blood cells and immune parameters show daytime variation in number and expression level [90]. The final dataset that was used in the analyses included only participants that had their time of blood draw between 7 am and 11 am, to limit the effect of diurnal variation being reflected in the DEGs. The cross-sectional design of the study limits causal inferences. Although we explored the effect of several factors that could influence the observed changes in gene expression, other factors (e.g., environmental factors) may also influence on the gene expression. We also had high number of missing data for BMI in all groups and for tobacco smoking in HC. Furthermore, we did not have WBC differential count to confirm changes in the blood cell composition.

The role of antipsychotic drug use on gene expression is challenging to examine in a cross-sectional setting. We identified some genes that were differently expressed between the main antipsychotic groups. However, gene expression might also be influenced by the duration of antipsychotic use and whether patients were on monotherapy or polytherapy as well as other factors. It should be noted that even if we did not find any significant differences between the main antipsychotic groups for the immature neutrophil genes, we cannot rule out that certain types of antipsychotic drugs and the duration of treatment might influence the gene transcription.

## Conclusion

This dataset constitutes the largest cohort study of whole blood transcriptomes to date from patients with SCZ or BD. We show that the gene expression pattern in both SCZ and BD has a strong signature of immature neutrophils with association to lipid changes, indicating alterations in the innate immune system in these patient groups. Future work should aim to further elucidate the role of neutrophils in psychosis, antipsychotic response, and associated comorbidities with the aim to translate findings into clinical practice.

## Supplementary information

Supplementary methods and results

Meta analysis

Differential expression analysis

Sex effect on gene expression

Correlation analysis

Smoking related differential expression analysis

Symptoms correlation analysis
