# Supplementary material for: Patients with schizophrenia and bipolar disorder display a similar global gene expression signature in whole blood that reflects elevated proportion of immature neutrophil cells with association to lipid changes
Source: Transl Psychiatry. 2023 May 5;13:147. doi: 10.1038/s41398-023-02442-1 (PMC10163263; doi:10.1038/s41398-023-02442-1)
Supplement: Supplementary file 1 — Supplementary methods and results [file 41398_2023_2442_MOESM1_ESM.docx]

**Supplementary Methods and Results**

*Microarray data processing and quality control*

The RNA samples were run in two batches (1195 samples in the first batch and 696 samples in the second batch), based on availability of the samples. The results were highly similar in the two runs as confirmed by the hypergeometric overlap (p-value < 3E-09). We used the meta-analysis tool METAL^1^ to compare the gene lists with p-values from the two batches (Table S1). After having verified the high similarity between the two datasets, all samples were merged into one common dataset to obtain larger groups. The combined dataset consisted of 1891 samples (657 SCZ, 375 BD, 177 other diagnoses, and 682 HC). Figure S1 shows a flow chart of the sample processing and filtering of samples to obtain the final dataset used in this study with 721 samples (329 SCZ, 203 BD, and 189 HC).


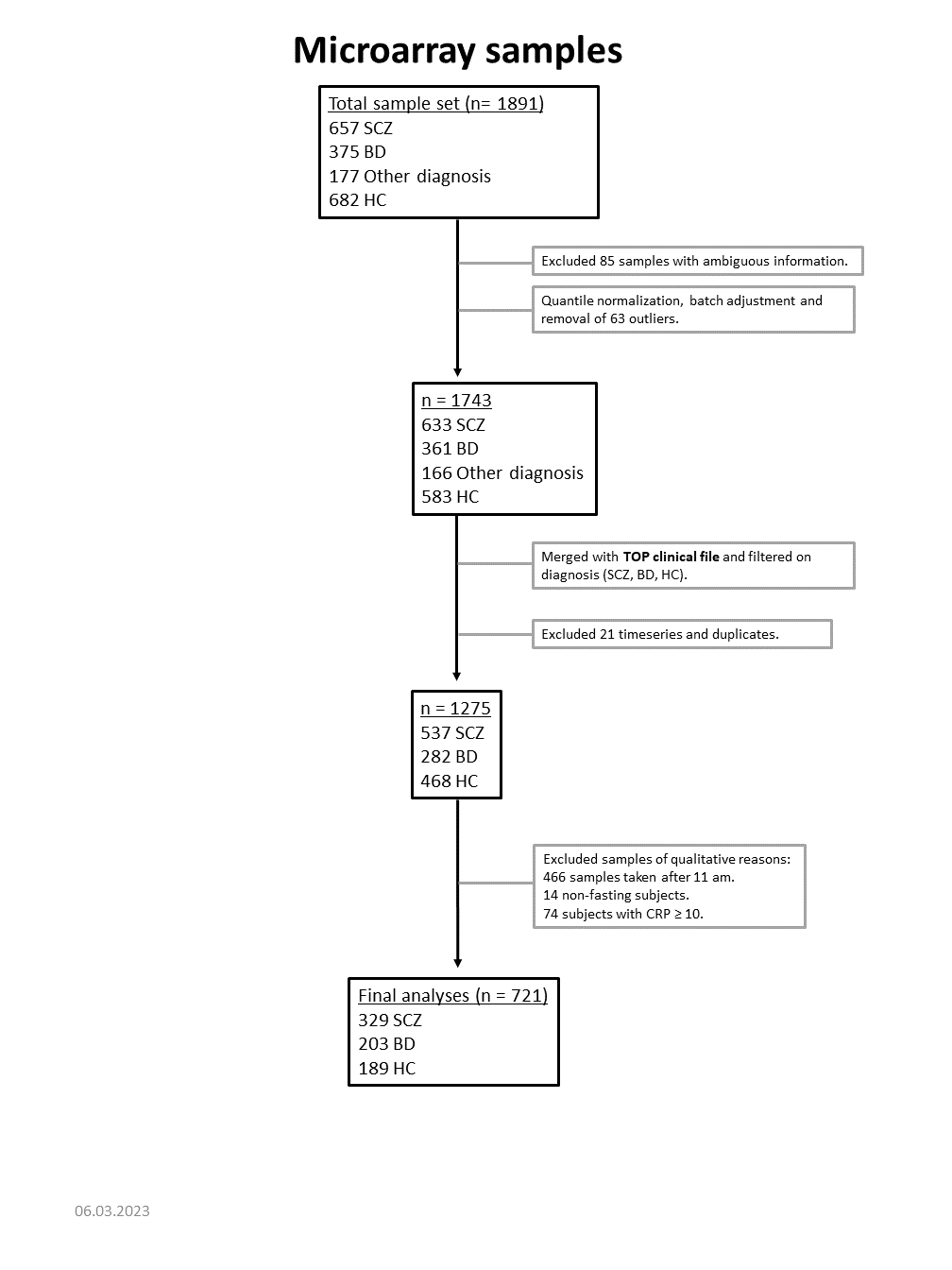


Figure S1: Flow chart of microarray sample processing and quality control.

*Cell deconvolution by CIBERSORTx*

We used CIBERSORTx ([www.cibersortx.stanford.edu](http://www.cibersortx.stanford.edu)) to estimate cell proportions based on gene expression values^5^. The leukocyte gene signature matrix LM22 was used as the reference dataset, allowing deconvolution of 22 blood cell subsets. Unfortunately, LM22 does not include our cell type of interest, *i.e*., the immature neutrophils, and to our knowledge, such reference data sets are not yet available. With this limitation, we calculated the proportion of the 22 blood cell subsets based on our gene expression data. Samples with non-significant p-value (> 0.05) for the deconvolution were excluded before comparing phenotypic groups with pairwise Wilcox test.

The cell deconvolution suggests lower levels of resting NK cells and activated CD4 memory T-cells in SCZ and BD vs HC and higher levels of naïve B-cells, regulatory T-cells (Tregs), and activated dendritic cells (DC). Resting CD4 memory T-cells were only significant in BD vs HC, but not in SCZ. (Figure S2).


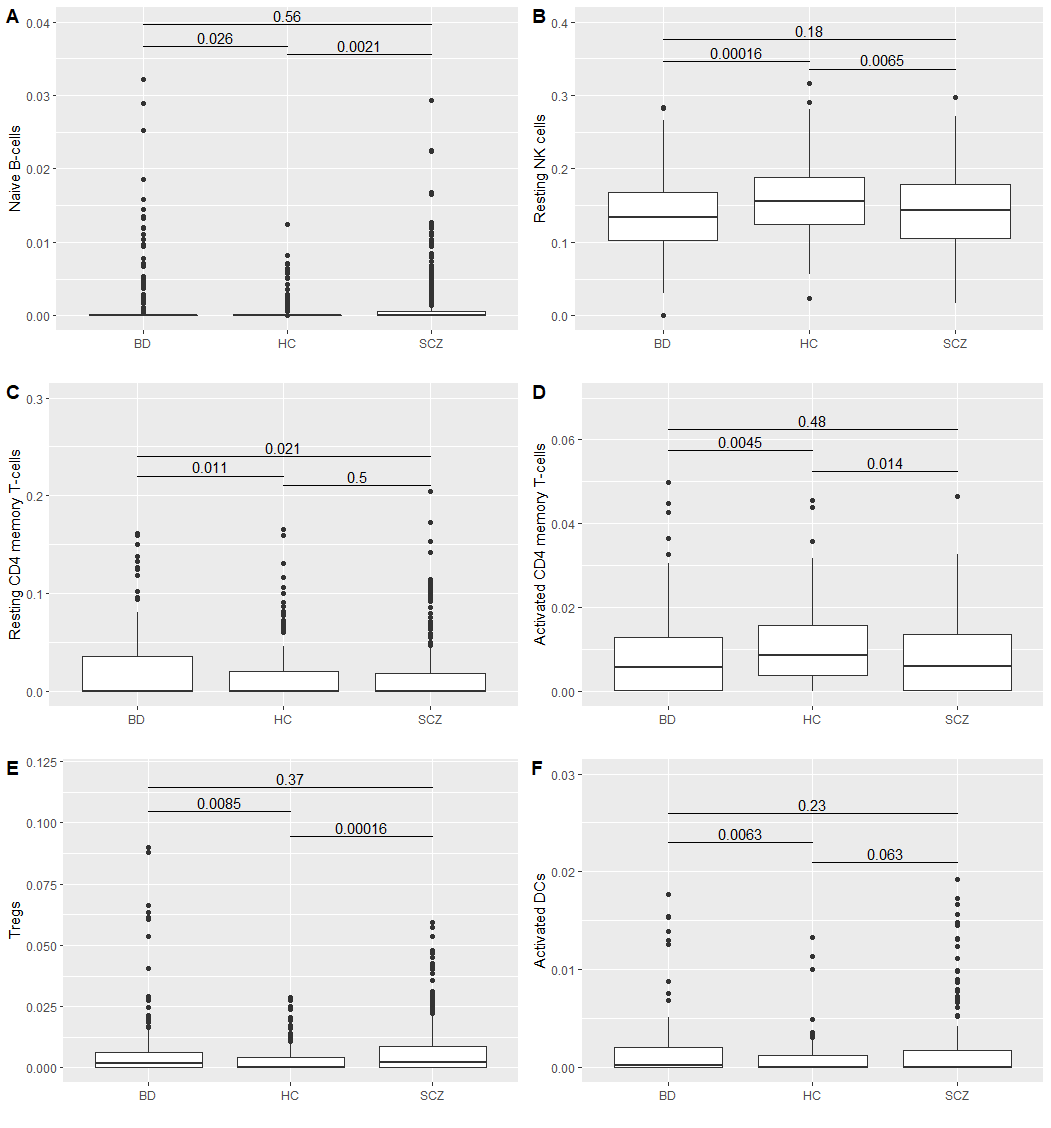


**Figure S2**: Cell proportions of cell types that showed significant differences in SCZ and BD compared to HC: A) Naïve B-cells, B) Resting NK cells, C) Resting CD4 memory T-cells, D) Activated CD4 memory T-cells, E) Tregs, and F) Activated DCs. Significance is annotated with the ggsignif package.

*Additional figures:*


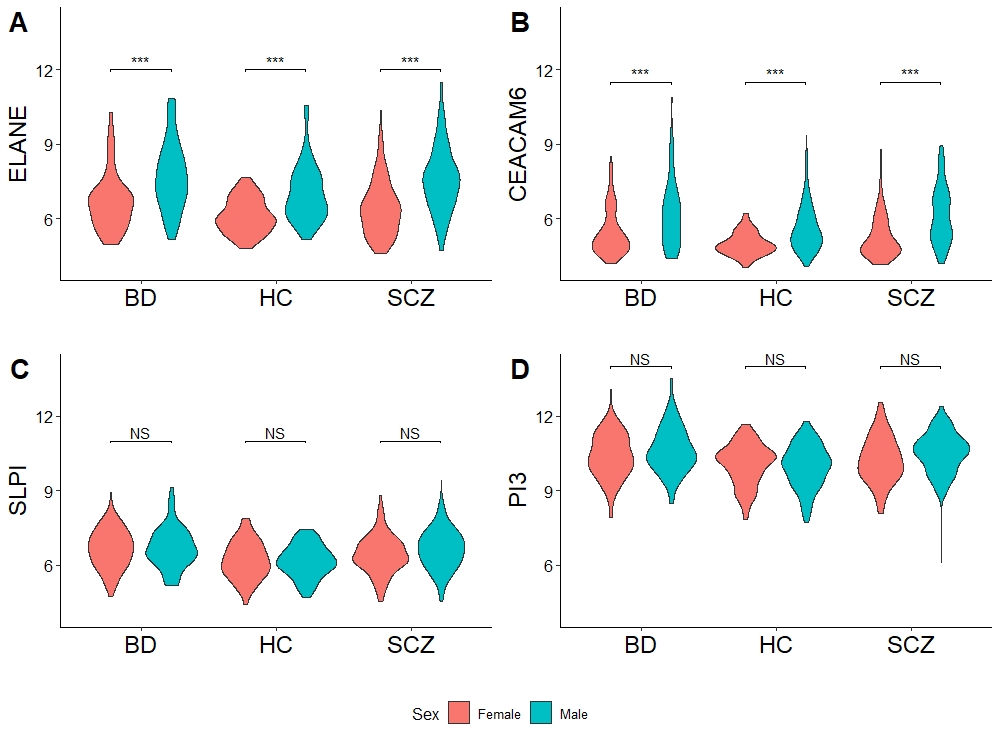


**Figure S3:** **Sex-dependent gene expression.** Violin plots (combining box plot and kernel density plot) of the expression level of four selected genes (*ELANE, CEACAM6, SLPI,* and *PI3*) in males (green) compared to females (red). The violin plot describes the distribution of expression signals for all participants in the various groups (HC/BD/SCZ and male/female). Normalized gene expression signals are log2 transformed. Significance is annotated with the ggsignif package. Significance level is indicated with *** = p-value = < 0.001, NS = not significant.


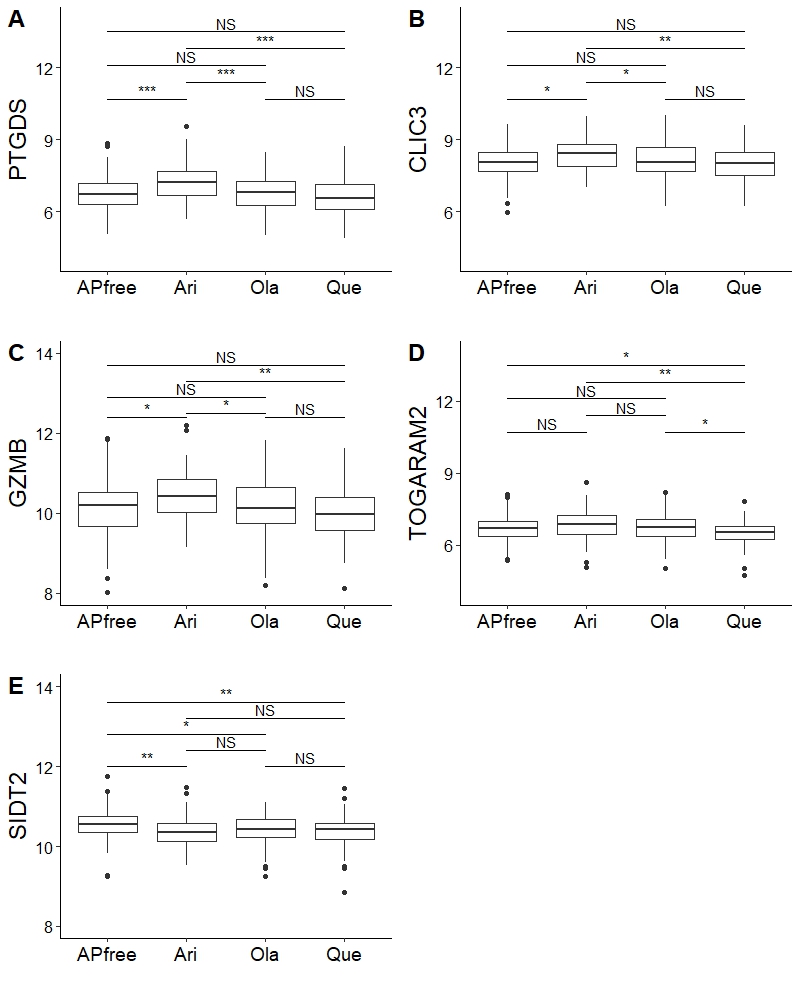


**Figure S4: Differences in gene expression between antipsychotic groups.** A-C: Levels of *PTGDS, CLIC3*, and *GZMB* are significantly higher in aripiprazole users compared to antipsychotic non-users and olanzapine or quetiapine users. D: *TOGARAM2* is lower in quetiapine users compared to the other groups. E: *SIDT2* is higher in antipsychotic non-users compared to antipsychotic users. APfree = Antipsychotic-free, Ari = aripiprazole, Ola = olanzapine, and Que = quetiapine. Normalized gene expression signals are log2 transformed. Significance is annotated with the ggsignif package. Significance level is indicated with * = p-value < 0.05, ** = p-value < 0.01 and *** = p-value = < 0.001. NS = not significant.

**Table S2: Statistical calculation of differences between antipsychotic groups.**

| **Gene** | **group1** | **group2** | **df** | **t-statistic** | **p** | **p.adj** |
| --- | --- | --- | --- | --- | --- | --- |
| **CLIC3** | Ari | APfree | 416 | 2.78 | 0.0057 | 0.0170 |
| **CLIC3** | Ari | Ola | 416 | 2.53 | 0.0118 | 0.0236 |
| **CLIC3** | Ari | Que | 416 | 3.35 | 0.0009 | 0.0053 |
| **GZMB** | Ari | APfree | 416 | 2.56 | 0.0107 | 0.0259 |
| **GZMB** | Ari | Ola | 416 | 2.50 | 0.0129 | 0.0259 |
| **GZMB** | Ari | Que | 416 | 3.69 | 0.0003 | 0.0016 |
| **PTGDS** | Ari | APfree | 416 | 3.53 | 0.0005 | 0.0009 |
| **PTGDS** | Ari | Ola | 416 | 3.67 | 0.0003 | 0.0008 |
| **PTGDS** | Ari | Que | 416 | 4.43 | 0.0000 | 0.0001 |
| **SIDT2** | APfree | Ari | 416 | 3.06 | 0.0023 | 0.0070 |
| **SIDT2** | APfree | Ola | 416 | 2.77 | 0.0058 | 0.0117 |
| **SIDT2** | APfree | Que | 416 | 3.34 | 0.0009 | 0.0055 |
| **TOGARAM2** | Que | APfree | 416 | -2.58 | 0.0103 | 0.0206 |
| **TOGARAM2** | Que | Ari | 416 | -3.43 | 0.0007 | 0.0040 |
| **TOGARAM2** | Que | Ola | 416 | -2.88 | 0.0042 | 0.0127 |

**References:**

1 Willer CJ, Li Y, Abecasis GR. METAL: fast and efficient meta-analysis of genomewide association scans. *Bioinforma Appl NOTE* 2010; **26**: 2190–2191.

2 Schmittgen TD, Livak KJ. Analyzing real-time PCR data by the comparative C(T) method. *Nat Protoc* 2008; **3**: 1101–1108.

3 Linzmeier RM, Ganz T. Human defensin gene copy number polymorphisms: comprehensive analysis of independent variation in alpha- and beta-defensin regions at 8p22-p23. *Genomics* 2005; **86**: 423–30.

4 Etienne G, Dupouy M, Costaglioli P, Chollet C, Lagarde V, Pasquet J-M *et al.* α-defensin 1-3 and α-defensin 4 as predictive markers of imatinib resistance and relapse in CML patients. *Dis Markers* 2011; **30**: 221–227.

5 Newman AM, Steen CB, Liu CL, Gentles AJ, Chaudhuri AA, Scherer F *et al.* Determining cell type abundance and expression from bulk tissues with digital cytometry. *Nat Biotechnol* 2019; **37**: 773–782.
